# Initial Influenza Virus Replication Can Be Limited in Allergic Asthma Through Rapid Induction of Type III Interferons in Respiratory Epithelium

**DOI:** 10.3389/fimmu.2018.00986

**Published:** 2018-05-17

**Authors:** Sujin An, Yung Jin Jeon, Ara Jo, Hyun Jung Lim, Young Eun Han, Sung Woo Cho, Hye Young Kim, Hyun Jik Kim

**Affiliations:** ^1^Department of Otorhinolaryngology, Seoul National University College of Medicine, Seoul, South Korea; ^2^Seoul National University Hospital, Seoul, South Korea; ^3^The Laboratory of Mucosal Immunology, Department of Biomedical Sciences, Seoul National University College of Medicine, Institute of Allergy and Clinical Immunology, Seoul National University Medical Research Center, Seoul, South Korea

**Keywords:** influenza A virus, type III interferon, Th2 cytokines, asthma, acute viral lung infection

## Abstract

Although asthmatics has been considered to be highly susceptible to respiratory viral infection and most studies have focused on exacerbation of asthma by influenza A virus (IAV) infection, few experimental evidences exist to directly demonstrate that asthmatic mice are actually resistant to IAV infection. Here, we show that asthmatic mice are not highly susceptible to IAV in the early stage of infection and type III interferon (IFN) maintains antiviral immune response in the lung of IAV-infected asthmatic mouse resulting in inhibition of initial viral spread. C57BL/6 mice with allergic asthma were infected with IAV (WS/33: H1N1) and survival rate, body weight, viral titer, histopathological findings of lung and cytokine profiles including IFNs and Th2 cytokines were measured. Notably, asthmatic mice were significantly resistant to IAV and showed lower viral load until 7 days after infection. Furthermore, IAV-infected asthmatic mice exhibited decreased Th2-related inflammation in lung tissue until 7 days. These increased antiviral resistant mechanism and reduced Th2 inflammation were attributable to rapid induction of type III IFNs and blockade of type III IFNs in asthmatic lung led to aggravated IAV infection and to enhance the production of Th2 cytokines. Asthmatic mice showed bi-phasic responses against IAV-caused lung infection such as rapid production of type III IFNs and subsequent induction of type II IFNs. Actually, IAV-infected asthmatic mice become vulnerable to IAV infection after 7 days with noticeable morbidity and severe weight loss. However, intranasal administration of type III IFNs protects completely asthmatic mice from IAV-mediated immunopathology and lung infection until 14 days after infection. Taken together, our study indicates that the rapid induction of type III IFN might be distinctive immunological findings in the respiratory tract of IAV-infected asthmatic mice at the early stage of infection and crucial for suppression of initial viral spread *in vivo* asthma accompanying with restriction of Th2 cytokine productions.

## Introduction

Allergic asthma is caused by sensitization to innocuous allergens *via* airway exposure. This type of asthma is thought to arise from an imbalance in T helper type I (Th1)-Th2 immune regulation, resulting in increased levels of the Th2 cytokines interleukin (IL)-4, IL-5, and IL-13, which have been proven to be important drivers of allergic airway inflammation in asthma (1, 2). Asthma exacerbations are acute attacks of asthma, accompanied by sudden decrease of lung function, most often precipitated by a respiratory viral infection and are responsible for the vast majority of the mortality associated with asthma. Although adequate control of asthma has been achieved, more appropriate controls for respiratory viral infection are needed to reduce acute exacerbation of respiratory symptoms in asthmatics (3, 4).

The innate immune system of the respiratory epithelium serves as the first line of antiviral defense against invading respiratory viruses including influenza A virus (IAV) (5). Traditionally, the antiviral innate immune response has been thought to be exclusively mediated by type I interferons (IFNs) followed by the adaptive immune response (5, 6). However, emerging evidence indicates that type III IFN is likely to be mainly required for immune responses in respiratory tract. In particular, type III IFN has been shown to be dominant IFN which is produced in respiratory tract against respiratory viral infection and provide front-line protection against respiratory virus to suppress initial viral spread in respiratory epithelium (7, 8). Moreover, type III IFN-mediated innate immune response is necessary to protect the lungs from IAV infection beyond antiviral properties of type I IFNs (9).

Impaired innate immune responses have been reported to be potentially responsible for the increased susceptibility to infections in asthmatics. Moreover, dysregulation of antiviral immune responses related to Th2 cytokines has been suggested to explain the higher susceptibility of the asthmatic respiratory epithelium to viral infection (10–12). Strong links between low expression of type III IFNs, severity of allergic asthma, and asthma exacerbations have been described (13). In the absence of detectable viral infection, asthmatic patients with active disease still exhibit an inverse correlation between type III IFN level and the severity of allergic response in the airways (12). However, some studies have also investigated whether asthmatic mice are more resistant to respiratory viral infection and which bystander immune mechanisms are activated during the induction of asthma and contribute to protecting the asthmatics against respiratory viral infection (14, 15).

To address the antiviral resistance of IAV-infected asthmatic mice, we sought to determine first if the asthmatic mice are more susceptible or resistant to IAV infection. We found that the asthmatic mice were not highly susceptible to IAV infection and type III IFNs were preferentially induced in the lung of IAV-infected asthmatic mouse at early stage of infection. Rapid induction of type III IFNs led to accelerated clearance of IAV, accompanied by increased type II IFN secretion. In addition, intranasal administration of type III IFNs provided more potent antiviral resistance to IAV-infected asthmatic mice. This study suggest that a better understanding about the role of type III IFNs in this asthmatic airway need to be achieved to prevent higher viral loads in the asthmatic airway and it can provide new insights into strategies for reducing asthmatic exacerbation from respiratory viral infection.

## Materials and Methods

### Allergen Sensitization and Challenge Protocol

C57BL/6J (B6) mice (Orientalbio, Seoul, Korea) aged 7 weeks (19–23 g) were used for the development of non-asthmatic and asthmatic mice. Asthma was induced by first sensitizing male B6 mice intraperitoneally (i.p.) with OVA in aluminum hydroxide and then challenging intranasally (i.n.) with soluble OVA (OVA/OVA). Phosphate-buffered saline (PBS)-challenged mice (OVA/PBS) (hereafter referred to as non-asthmatic mice) were used as a negative control. B6 mice were sensitized with two intraperitoneal injections on days 0 and 14 of 7.5 μg OVA (Grade V; Sigma, MO, USA) complexed with aluminum hydroxide as adjuvant (Sigma, MO, USA). On days 21, 22, 23, and 24, mice were challenged intranasally with 7.5 g OVA mixed with PBS (OVA/OVA). Control mice received an intraperitoneal injection of OVA at the same concentration and were challenged with PBS alone by intranasal inoculation (OVA/PBS). Airway hyper-responsiveness (AHR) was measured in anesthetized mechanically ventilated B6 mice (Flexivent ventilator, SciReq, Montreal, QC, Canada) at 24 h after the last intranasal OVA exposure. AHR was measured invasively using a body plethysmograph (Buxco Electronics, Inc., Wilmington, NC, USA).

### Mice and Virus Inoculation

Influenza A virus (WS/33: H1N1, ATCC, Manassas, VA, USA) was used to induce acute viral lung infection. Virus stocks were grown in Madin–Darby canine kidney cells in virus growth medium according to a standard procedure (16). Briefly, after 48 h of incubation at 37°C, the supernatants were harvested and centrifuged at 5,000 rpm for 30 min to remove cellular debris. Virus stocks were titrated on MDCK cells using a tissue culture infectious dose assay and stored at −80°C. The B6 mice used in the study, like other commercially available strains of inbred mice, carry a dysfunctional Mx1 gene and are not congenic B6 mice with a functional Mx1 gene, which are derived from influenza-resistant mice.

For viral infections, IAV (WS/33, H1N1; 213 pfu in 30 µl PBS) were inoculated into WT mice by intranasal delivery and asthmatic mice (OVA/OVA) were also infected with IAV at 25 days after first sensitization. Mice were euthanized at the end of each experiment with overdose of tiletamine/zolazepam (5 mg) and xylazine (0.23 mg) and after euthanizing, bronchoalveolar lavage (BAL) fluid was obtained from the lungs by lavaging with 1,000 µl 0.5 mM ethylene diamine tetraacetic acid in PBS after cannulation of the trachea. The BAL fluid was used for enzyme-linked immunosorbent assay (ELISA) for measuring secreted protein levels and plaque assay to determine the viral titer. Mouse lung tissue was also harvested for real-time polymerase chain reaction (PCR), microarray, and immunohistochemistry analyses.

### Real-Time PCR

Lung tissue was obtained from mice infected with WS/33 (H1N1) on 1, 3, 5, 7, 10, and 14 days postinfection, after which total RNA was isolated using TRIzol (Invitrogen). cDNA was synthesized from 3 µg of RNA with random hexamer primers and Moloney murine leukemia virus reverse transcriptase (Perkin Elmer Life Sciences, Waltham, MA, USA and Roche Applied Science, Indianapolis, IN, USA). Amplification was performed using the TaqMan Universal PCR Master Mix (PE Biosystems, Foster City, CA, USA) according to the manufacturer’s protocol. Briefly, amplification reactions had a total volume of 12 µl and contained 2 µl of cDNA (reverse transcription mixture), oligonucleotide primers (final concentration of 800 nM), and TaqMan hybridization probe (200 nM). Real-time PCR probes were labeled at the 5′ end with carboxyfluorescein (FAM) and at the 3′ end with the quencher carboxytetramethylrhodamine (TAMRA).

To quantify the intracellular levels of viral RNA and host gene expression levels, cellular RNA was used to generate cDNA. IAV level was monitored using quantitative PCR to amplify the *PA* gene (segment 3) with forward and reverse primers and probe 5′-ggccgactacactctcgatga-3′, 5′-tgtcttatggtgaatagcctggttt-3′, and 5′-agcagggctaggatc-3′, respectively. Primers for mouse IFN-α, IFN-β, IFN-λ_2/3_, and IFN-γ were purchased from Applied Biosystems (Foster City, CA, USA). Real-time PCR was performed using the PE Biosystems ABI PRISM^®^ 7700 Sequence Detection System. Thermocyling parameters were as follows: 50°C for 2 min, 95°C for 10 min, and then 40 cycles of 95°C for 15 s and 60°C for 1 min. Target mRNA levels were quantified using target-specific primer and probe sets for IAV WS/33 (H1N1), IFN-α, IFN-β, IFN-λ_2/3_, and IFN-γ. All PCR assays were quantitative and utilized plasmids containing the target gene sequences as standards. All reactions were performed in triplicate, and all real-time PCR data were normalized to the level of the housekeeping gene glyceraldehyde phosphate dehydrogenase (1 × 10^6^ copies) to correct for variation between samples.

### Quantification of Secreted Cytokines

The levels of secreted IFN-α (42120-1), IFN-β (42400-1), IFN-λ2/3 (DY1789B), and IFN-γ (DY485) were quantified using a Duoset ELISA kit (R&D Systems; Minneapolis, MN, USA) according to the manufacturer’s instructions for BAL fluid. This kit detects IFN-β, IL-28A, IL-28B, and IFN-γ. The working range of the assay was 62.5–4,000 pg/ml. The levels of secreted IL-4, IL-5, IL-6, and IL-13 were measured using a Luminex multiplex assay (R&D Systems) according to the manufacturer’s instructions.

### Inoculation With IFN-λ_2/3_-Neutralizing and IFN-γ-Neutralizing Antibodies

Specific neutralizing antibodies against IFN-λ_2/3_ and IFN-γ were used to functionally inhibit IFN-λ and IFN-γ in the mouse respiratory tract. Anti-IFN-λ_2/3_ (cat number: MAB1789) and anti-IFN-γ (cat number: MAB4851) neutralizing antibodies and isotype-control antibodies (rat IgG) were purchased from R&D Systems.

These antibodies were found to inhibit the secretion of IFN-λ_2/3_ and IFN-γ by more than 70% in BAL fluid. Neutralizing antibodies (10 μg/30 μl) were mixed with PBS and inoculated by intranasal delivery according to the manufacturer’s instructions (R&D Systems Inc.), concurrent with IAV infection. This procedure did not affect mouse viability. ELISA analysis confirmed that the neutralizing antibodies partially inhibited IFN-λ_2/3_ and IFN-γ secretion.

### Intranasal Delivery of Recombinant IFN-λ_2/3_

To determine whether IFN-λ_2/3_ controls acute IAV lung infection in our *in vivo* model, WT mice (*N* = 5) were administered recombinant IFN-λ_2/3_ *via* the intranasal route in a total volume of 30 μl PBS. The recombinant IFN-λ_2/3_ was purchased from Invitrogen (Carlsbad, CA, USA). IFN-λ_2_ and IFN-λ_3_ were mixed (IFN-λ_2_: 1 μg, IFN-λ_3_: 1 μg), and recombinant IFN-λ_2/3_ was inoculated into mice by intranasal delivery at the same time as IAV infection. IAV-infected mice were treated with mixed recombinant IFN-λ through the nasal cavity.

### Immunohistochemistry and Histological Analysis

Lung tissue was fixed in 10% (vol/vol) neutral buffered formalin and embedded in paraffin. Paraffin-embedded tissue slices were stained with hematoxylin/eosin (H&E) or periodic acid Schiff (PAS) solution (Sigma, Deisenhofen, Germany). Histopathological analysis of inflammatory cells in H&E-stained lung sections was performed in a blinded fashion using a semi-quantitative scoring system as previously described (13). Lung sections from at least five mice were examined. Briefly, peribronchiolar inflammation was scored as follows: 0, normal; 1, a few cells; 2, a ring of inflammatory cells one layer deep; 3, a ring of inflammatory cells two to four cells deep; and 4, a ring of inflammatory cells more than four cells deep (maximum score = 8). The histological score for PBS/PBS control mouse lung tissue was 0. At least six separate areas from similar sections within a single mouse were assessed, and at least five mice were assessed. The five best sections were used for evaluation. PMNs were counted by an examiner who was blinded to the experimental group; results are expressed as the number of cells per high power field.

### Plaque Assay

Virus samples were serially diluted with PBS. Confluent monolayers of MDCK cells in six-well plates were washed twice with PBS and then infected in duplicate with 250 μl/well of each virus dilution. The plates were incubated at 37°C for 45 min to facilitate virus adsorption. Following adsorption, a 1% agarose overlay in complete MEM supplemented with TPCK trypsin (1 µg/ml) and 1% fetal bovine serum was applied. The plates were incubated at 37°C, after which cells were fixed with 10% formalin at 2 days postinfection.

### Flow Cytometry

Single-cell suspensions were stained with the following monoclonal antibodies: Texas Red-anti-CD45 (Invitrogen), fluorescein isothiocyanate-anti-lineage cocktail (anti-CD3, anti-CD11c, anti-CD11b, anti-CD19, anti-CD49b, anti-F4/80, and anti-FcεRIα), Brilliant Violet 421-anti-Siglec-F (BD Biosciences), allophycocyanin (APC)-anti-CD11b, PE/Cy7-anit-CD90.2, and PE-anti-NK1.1 (BioLegend). All samples were blocked with 1 µg Fc block (from 2.4G2 ATCC HB-197) for 15 min before antibody staining at 4°C for 30 min in PBS containing 2% FCS (2% FCS-PBS). Cells were washed twice in 2% FCS-PBS, after which data were collected on a BD LSRFortessa X-20 cytometer (BD Biosciences). Data analysis was performed using FlowJo v10 10.1r1 (FlowJo, LLC, Ashland, OR, USA).

### Intracellular Cytokine Staining

For intracellular cytokine staining, single-cell suspensions were incubated in RPMI medium containing 10% FBS with PMA (100 ng/ml), ionomycin (1 µg/ml), and GolgiStop (BD) at 37°C for 4 h. After surface staining, the cells were fixed and permeabilized with a Fixation/Permeabilization Kit (eBioscience). Finally, the cells were stained with PE-anti-T-bet and APC-anti-IFN-γ antibodies (Biolegend). The respective isotype-control antibody was also used for each experiment.

### Statistical Analyses

Real-time PCR, plaque assay, and ELISA results are presented as median values (interquartile ranges for 25 and 75%). The statistical significance of differences between two groups was determined by the Mann–Whitney test. Histological scores were also evaluated by a non-parametric test (Wilcoxon rank sum test). All statistical analysis was performed with GraphPad Prism software (version 5; GraphPad Software, La Jolla, CA, USA). *p* Values <0.05 were considered to be statistically significant.

## Results

### OVA-Sensitized/Challenged C57BL/6 (B6) Mice Exhibit a Typical Asthmatic Phenotype

**Figure 1:**
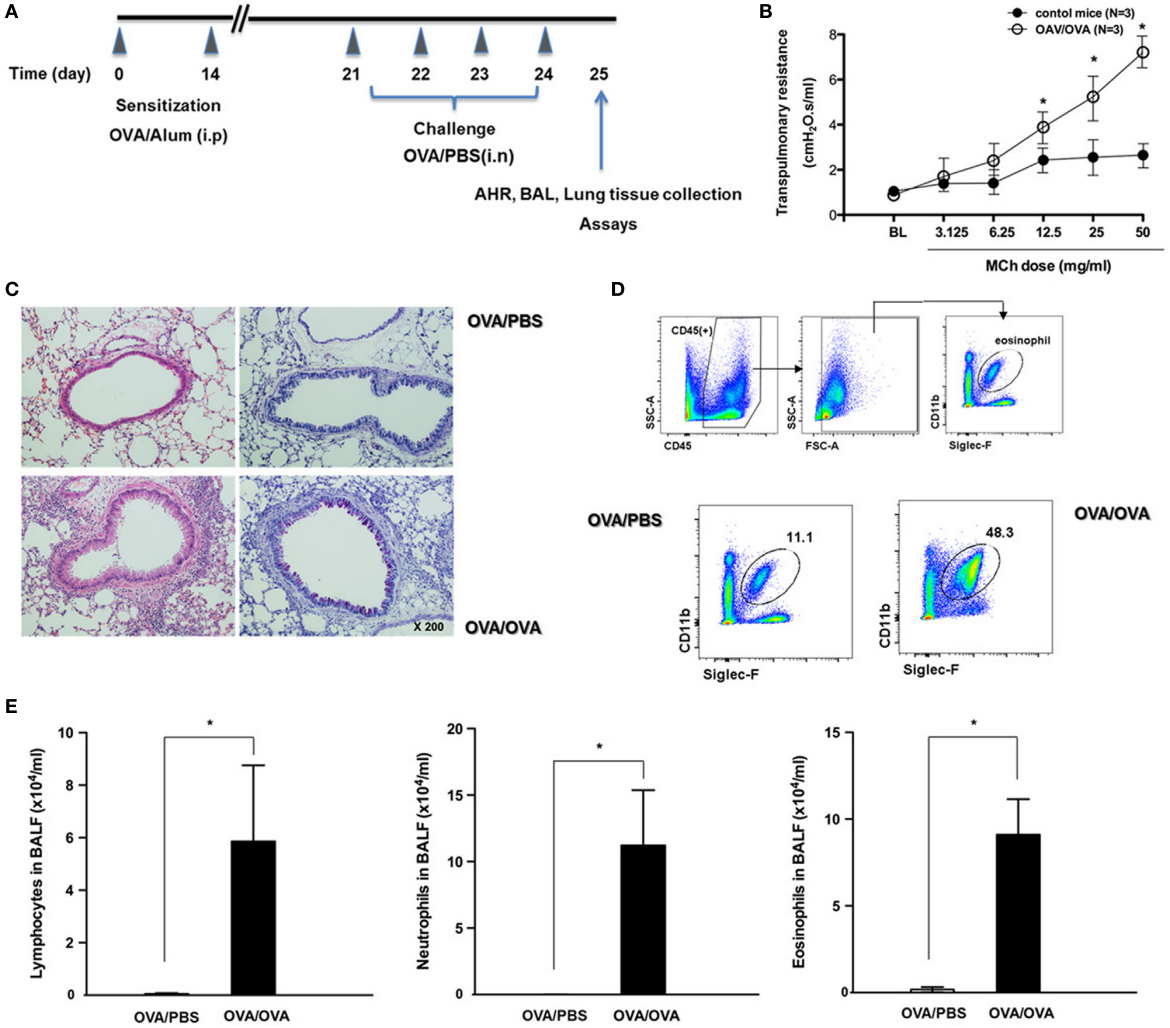
The development of allergic asthmatic mouse model. **(A)** The experimental protocol for development of allergic asthmatic mouse using C57BL/6. Non-asthmatic mice [OVA/phosphate-buffered saline (PBS), *N* = 5]. **(B)** Airway hyper-responsiveness was measured as methacholine-induced increases in transpulmonary resistance in mechanically ventilated mice. Data are expressed as mean value of percentage increased from base line of the transpulmonary resistance ± SD values of three mice (white dot: OVA/OVA, black dot: OVA/PBS). **(C)** Histological assessment of mucus secretion in asthmatic mice. Periodic acid Schiff-stained sections were assessed from five mice. **(D)** Flow cytometric analysis was done for isolation of eosinophils using anti-CD11c and anti-Siglec-F antibodies. **(E)** Bronchoalveolar lavage (BAL) fluid differential counts for lymphocytes, neutrophils, and eosinophils expressed as mean ± SD.

To assess the influence of allergic airway inflammation on susceptibility to respiratory viral infection, we developed an OVA-sensitized/challenged allergic asthma mouse model on a B6 genetic background (Figure 1A). Initially, AHR was measured in mice after inoculation of methacholine. Asthmatic mice (OVA/OVA) were observed to have a methacholine-induced increase in total lung resistance (*N* = 3, Figure 1B). As a complementary approach, H&E- and PAS-stained micrographs of lung sections were obtained from non-asthmatic (OVA/PBS) and asthmatic mice. Histological analysis revealed that the lungs of asthmatic mice were severely inflamed, with extensive inflammatory cell infiltration at the peribronchial areas of the lung. This infiltration was accompanied by significantly increased goblet cell metaplasia (Figure 1C). We found that the number of eosinophils was also increased in total lung tissue of asthmatic mice, and that the numbers of lymphocytes, neutrophils, and eosinophils were significantly increased in the BAL fluid of asthmatic mice (Figures 1D,E). Collectively, these findings demonstrate that an allergic asthma mouse model could be established with B6 mice, and that this model could be used to investigate the susceptibility of asthmatic mice to influenza virus infection.

### Asthmatic Mice Are Not Highly Susceptible to IAV Infection

**Figure 2:**
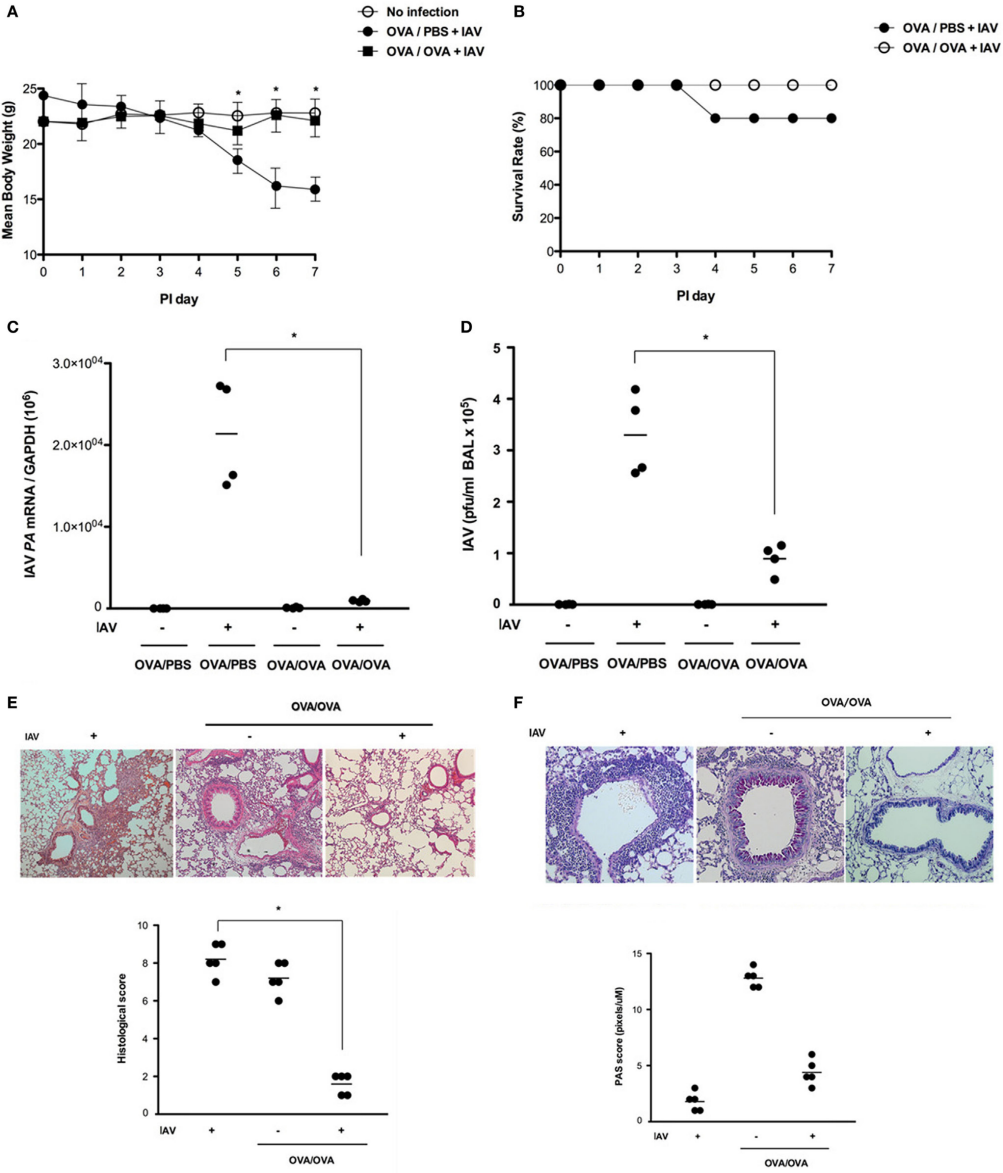

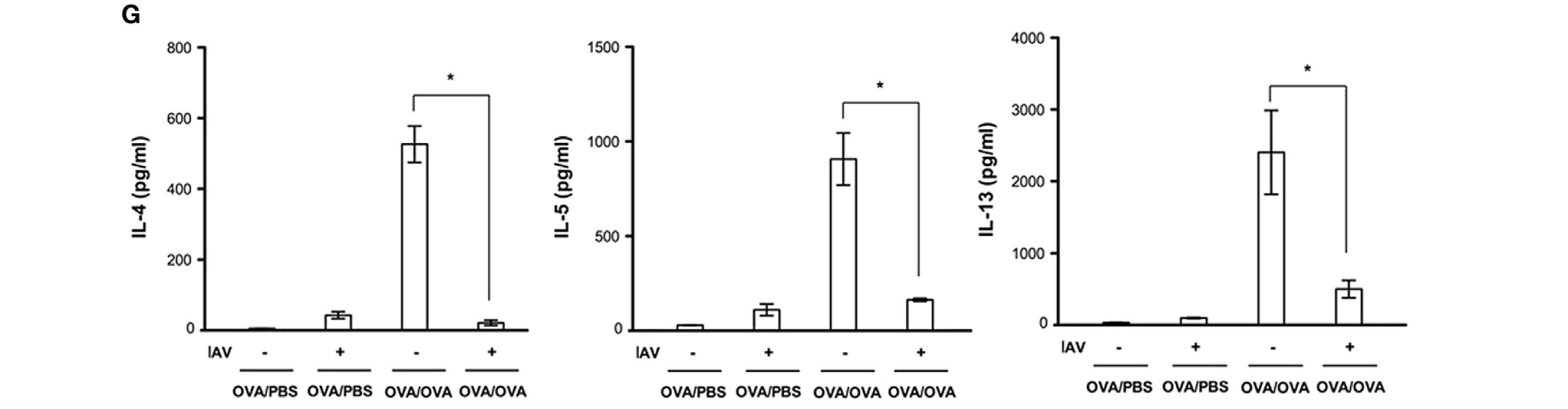
Kinetics of influenza A virus (IAV) infection in non-asthmatic and asthmatic mice. Non-asthmatic (*N* = 5) and asthmatic mice (*N* = 5) were infected with 213 pfu IAV WS/33 (H1N1), and body weight **(A)** and survival rate [**(B)**, *N* = 20] were assessed until 7 dpi. The IAV mRNA **(C)** and viral titer **(D)** in lung tissue and bronchoalveolar lavage (BAL) fluid, respectively, were determined at 7 dpi. Hematoxylin/eosin (H&E)-stained micrographs were also generated from lung sections obtained at 7 dpi **(E)**. Histological assessment for mucus secretion was performed on periodic acid Schiff (PAS)-stained lung sections of asthmatic mice **(F)**. A multiplex assay was performed to quantify the levels of secreted Th2 cytokines (i.e., IL-4, IL-5, and IL-13) in the BAL fluid of non-asthmatic and asthmatic mice **(G)**. Micrographs shown are representative of lung sections from five mice. Polymerase chain reaction, plaque assay, and multiplex assay results are presented as mean ± SD from five independent experiments (*p < 0.05 compared with the levels of non-asthmatic and asthmatic mice at 7 dpi).

Previously, we found that IAV-infected non-asthmatic mice exhibited a significant decrease in mean body weight and a decrease in body temperature, with the lowest drop at 7 dpi. They also showed 80% survival until 14 dpi (17). In this study, non-asthmatic mice (*N* = 5) and asthmatic mice (*N* = 5) were inoculated with WS/33 (H1N1) to determine the susceptibility of asthmatic mice to IAV lung infection. Then, the body weights and survival rates of non-asthmatic and asthmatic mice were compared until 7 dpi. Non-asthmatic mice showed significant weight loss from 5 dpi, and 20% of the mice died in the first 7 days after IAV infection. However, asthmatic mice did not exhibit significant weight loss or noticeable morbidity until 7 dpi, and all asthmatic mice survived the IAV infection (Figures 2A,B). The levels of IAV mRNA in non-asthmatic and asthmatic mice lung were next analyzed by real-time PCR, and the viral titers in the BAL fluid were measured by plaque assay at 7 dpi. The mean level of IAV mRNA and the mean viral titer were both significantly elevated at 7 dpi (mRNA level: 2.1 × 10^4^, viral titer: 3.3 × 10^5^ pfu/ml) in non-asthmatic mice. By contrast, the levels of IAV mRNA and viral titer were much lower in IAV-infected asthmatic mice (mRNA level: 1.4 × 10^3^, viral titer: 8.1 × 10^4^ pfu/ml, Figures 2C,D). As a complementary approach, lung sections were obtained from non-asthmatic and asthmatic mice at 7 dpi, and H&E-stained micrographs were generated. Histological analysis revealed severe subepithelial consolidation, peribronchial edema, and increased epithelium detachment in non-asthmatic mouse lung sections harvested at 7 dpi. The lungs of asthmatic mice were severely inflamed, with extensive inflammatory cell infiltration at the peribronchial areas without IAV infection. Notably, these histopathological findings were not detectable in the lung sections harvested from IAV-infected asthmatic mice at 7 dpi and the mean histological score was significantly lower in IAV-infected asthmatic mice (8.6 for the non-asthmatic mice vs. 1.8 for the asthmatic mice) (Figure 2E). This finding was accompanied by significantly increased goblet cell metaplasia in asthmatic mice, whereas remarkable reduction of goblet cells was observed in the respiratory epithelium of IAV-infected asthmatic mice at 7 dpi (13.2 in asthmatic mice vs. 4.6 in IAV-infected asthmatic mice, Figure 2F). In addition, AHR was measured after inoculation of methacholine in IAV-infected asthmatic mice at 7 dpi and a methacholine-induced increase in total lung resistance was not observed in IAV-infected asthmatic mice (Figure S1 in Supplementary Material).

A multiplex assay was next performed to quantify the levels of secreted Th2 cytokines, such as IL-4, IL-5, and IL-13, in the BAL fluid of asthmatic mice. While higher secretion of Th2 cytokines was observed at 7 dpi in asthmatic mice without IAV infection (IL-4: 532.3 ± 85.1 pg/ml, IL-5: 964.7 ± 98.5 ng/ml, IL-13: 2,487.5 ± 765.5 ng/ml), cytokine secretion was significantly attenuated in IAV-infected asthmatic mice (IL-4: 64.2 ± 11.5 pg/ml, IL-5: 121.7 ± 32.6 ng/ml, IL-13: 498.5 ± 104.8 ng/ml, Figure 2G). These results indicate that asthmatic mice were not highly susceptible to IAV infection. Moreover, IAV-infected asthmatic mice exhibited minimal induction of Th2 cytokine secretion accompanying decreased asthma-related histopathological findings, including goblet cell hyperplasia.

### Asthmatic Mice Maintain Intact IFN-Related Innate Immunity After IAV Infection

**Figure 3:**
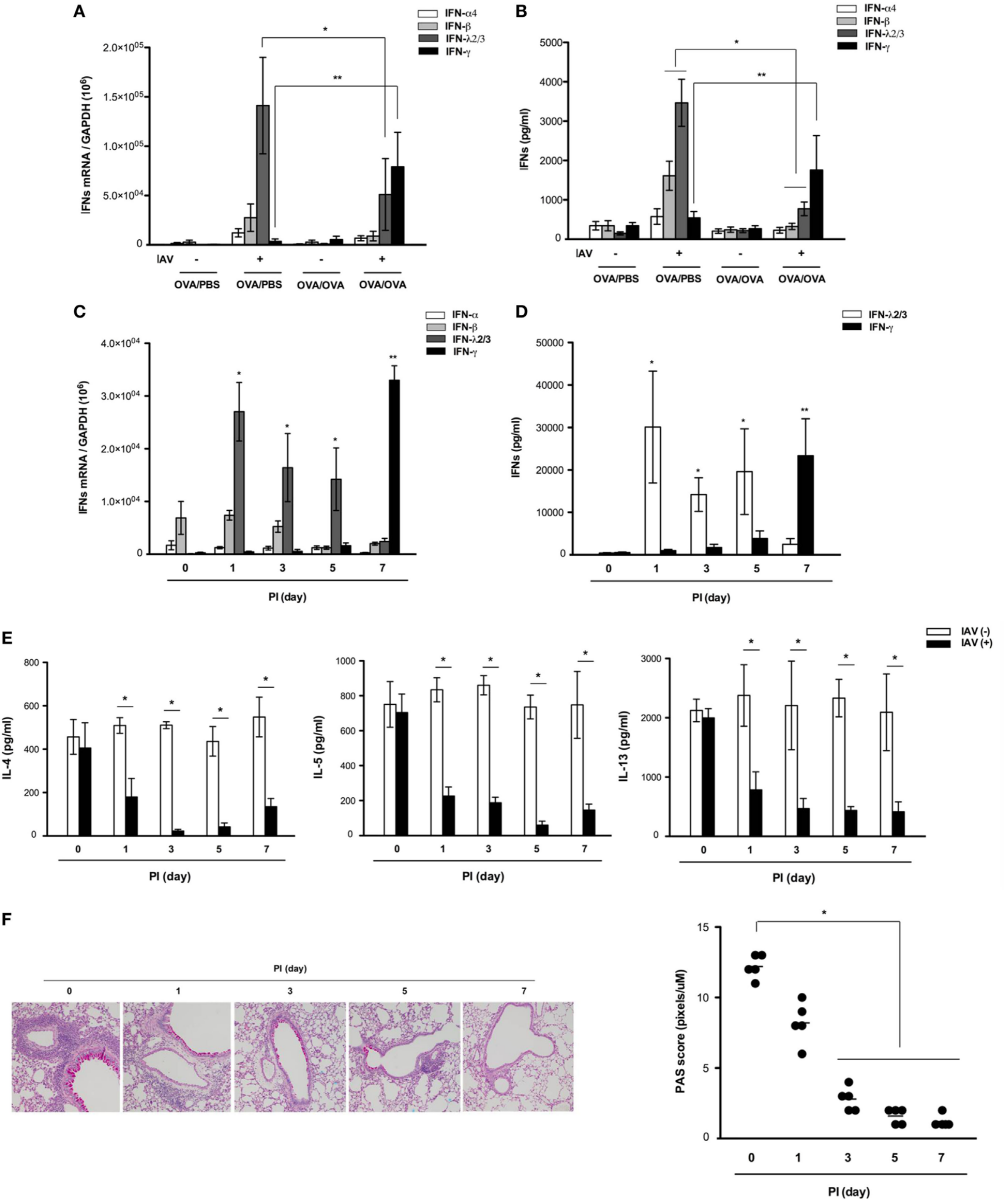
Expression and secretion of various interferons (IFNs) after influenza A virus (IAV) infection of non-asthmatic and asthmatic mice. Non-asthmatic (*N* = 5) and asthmatic mice (*N* = 5) were infected with 213 pfu IAV WS/33 (H1N1). The mRNA levels in the lung **(A)** and levels of secreted IFN-α, IFN-β, IFN-λ_2/3_, and IFN-γ in bronchoalveolar lavage (BAL) fluid **(B)** were determined by real-time polymerase chain reaction (PCR) and enzyme-linked immunosorbent assay, respectively, at 7 days after infection. The mRNA levels and levels of secreted IFN-α, IFN-β, IFN-λ_2/3_, and IFN-γ in the lung **(C)** and BAL fluid **(D)** were determined in IAV-infected asthmatic mice until 7 dpi. A multiplex assay was performed to quantify the levels of secreted Th2 cytokines in the BAL fluid of IAV-infected asthmatic mice until 7 dpi **(E)**. Histological assessment was performed using periodic acid Schiff (PAS)-stained lung sections of asthmatic mice (*N* = 5) **(F)** harvested at 0 (*N* = 5), 1 (*N* = 5), 3 (*N* = 5), 5 (*N* = 5), and 7 (*N* = 5) dpi. Micrographs shown are representative of five mice. PCR, plaque assay, and multiplex assay results are presented as mean ± SD from five independent experiments (^*,**^p < 0.05 compared with the levels in non-asthmatic and asthmatic mice at 7 dpi, Figures 2B–D *IFN-λ_2/3_, **IFN-γ).

To assess whether the increased antiviral resistance of asthmatic mice was due to an increased IFN-related immune response, we investigated the influence of asthma status on induction of IFN from the respiratory tract after IAV infection. Real-time PCR analysis revealed that the mRNA levels of IFN-α_4_, IFN-β, and IFN-λ_2/3_ were higher at day 7 after IAV infection in lung tissue from non-asthmatic mice (IFN-α4: 1.2 × 10^4^ ± 3.4 × 10^3^, IFN-β: 2.8 × 10^4^ ± 4.3 × 10^3^, IFN-λ_2/3_:1.4 × 10^5^ ± 5.2 × 10^4^, Figure 3A) but minimal induction of IFN mRNA, especially IFN-λ_2/3_, was observed in lung tissue from IAV-infected asthmatic mice (IFN-λ_2/3_:4.8 × 10^4^ ± 1.4 × 10^4^). Next, ELISA was performed to quantify the levels of secreted IFNs in the BAL fluid after IAV infection. The levels of secreted IFN-β and IFN-λ_2/3_ were all increased at day 7 after IAV infection in the BAL fluid of non-asthmatic mice (IFN-β: 1,514.5 ± 387.7 pg/ml, IFN-λ_2/3_: 3,298.6 ± 869.4 pg/ml), but IAV-infected asthmatic mice exhibited impaired secretion of IFN-β and IFN-λ_2/3_ at 7 dpi (IFN-β: 219.5 ± 47.6 ng/ml, IFN-λ_2/3_: 683.4 ± 97.7 ng/ml) (Figure 3B). However, significantly higher induction of IFN-γ mRNA (6.4 × 10^4^ ± 1.4 × 10^4^) and protein secretion (1,865.7 ± 731.4 pg/ml) were observed at 7 dpi in the lung tissue and BAL fluid of IAV-infected asthmatic mice compared with non-asthmatic mice (mRNA: 2.4 × 10^3^ ± 5.4 × 10^2^, protein: 286.3 ± 48.7 pg/ml). This finding suggests that the IFN-γ production in IAV-infected asthmatic mice observed at 7 dpi might be critically involved in resistance to IAV infection and decreased secretion of Th2 cytokines in the asthmatic respiratory tract.

While neither IFN-β nor IFN-λ_2/3_ was induced in the respiratory tract of IAV-infected asthmatic mice at 7 days after infection, we next analyzed the levels of these IFNs in asthmatic mice until 7 days after infection to determine if rapid and transient alterations occurred. To this end, asthmatic mice were infected with IAV WS/33 (H1N1) *via* the intranasal route (213 pfu/30 μl), and the production of IFNs and Th2 cytokines was measured at 0, 1, 3, 5, and 7 dpi.

Real-time PCR and ELISA analyses revealed that induction of IFN-γ mRNA and secretion of IFN-γ did not occur until 5 dpi (3.6 × 10^4^ ± 6.5 × 10^3^ and 2,482.5 ± 587.3 pg/ml, respectively) in IAV-infected asthmatic mice, with the highest levels exhibited at 7 dpi. By contrast, transcription (2.8 × 10^4^ ± 4.6 × 10^3^) and secretion of IFN-λ_2/3_ (3,002.4 ± 1,284.9 pg/ml) were elevated most significantly at 1 dpi compared with the mRNA levels of IFN-α and -β; these levels gradually decreased until 7 dpi (Figures 3C,D). This rapid production of IFN-λ_2/3_ was accompanied by significant reduction of IL-4, IL-5, and IL-13 in the BAL fluid of IAV-infected asthmatic mice until 7 dpi (Figure 3E). Goblet cell metaplasia and hyper-secretion of airway mucus appeared to be significantly reduced from 3 dpi; moreover, PAS score of asthmatic mice were lowest at 7 days after IAV infection (PI 0d: 12.7, PI 1d: 7.8, PI 3d: 2.6, PI 5d: 1.4, PI 7d: 1.3, Figure 3F).

These findings show that IFN-λ_2/3_ and IFN-γ were both markedly induced, and that IFN-λ_2/3_ induction was driven rapidly after IAV infection in the lungs of IAV-infected asthmatic mice than IFN-γ. This induction appears to reduce Th2 cytokine secretion in asthmatic mice after infection, which might be required for the enhanced resistance of asthmatic mice to IAV replication.

### Rapid Induction of IFN-λ Limits IAV Replication in the Lungs of Asthmatic Mice and Controls IFN-γ Secretion From Respiratory Epithelial Cells

**Figure 4:**
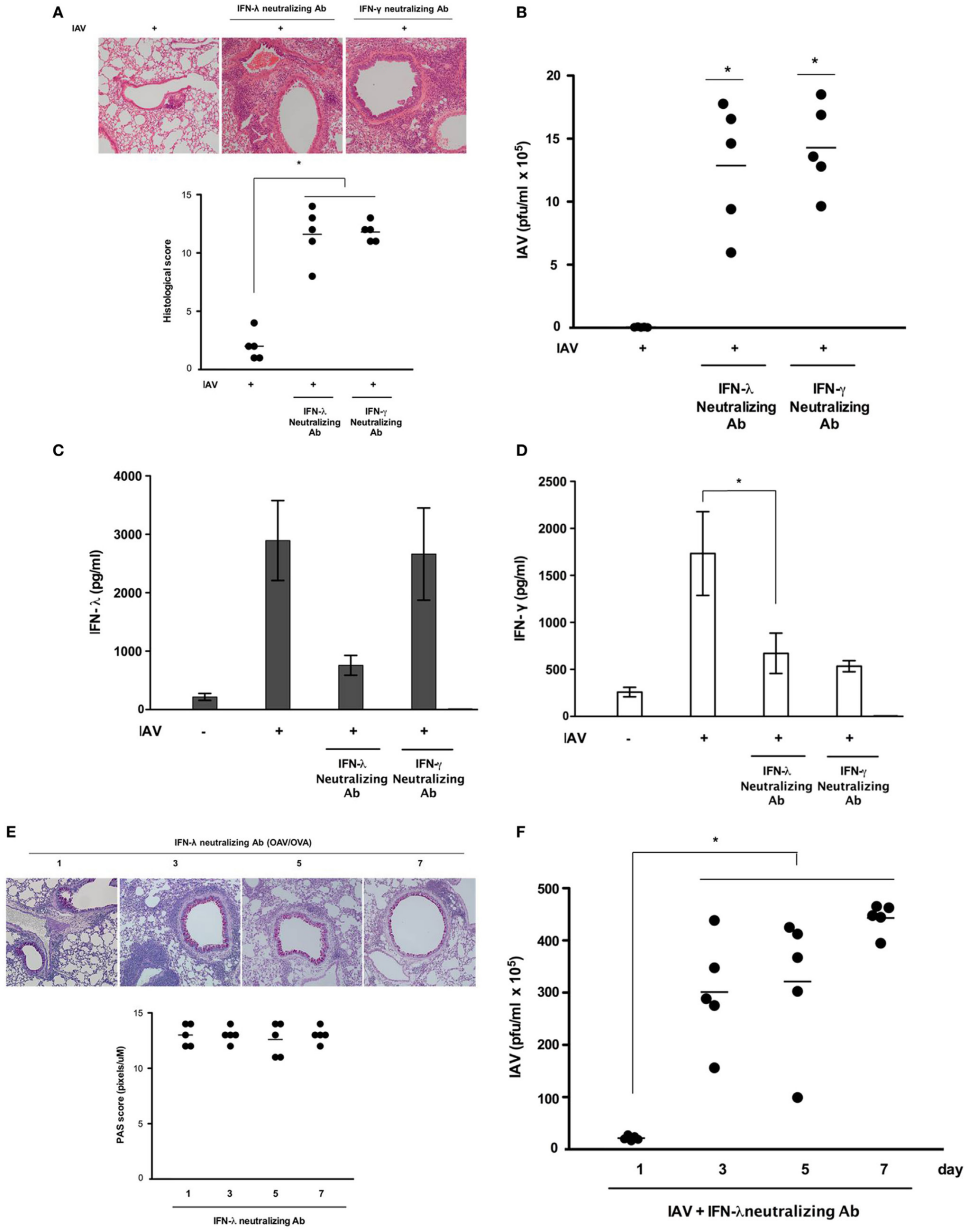

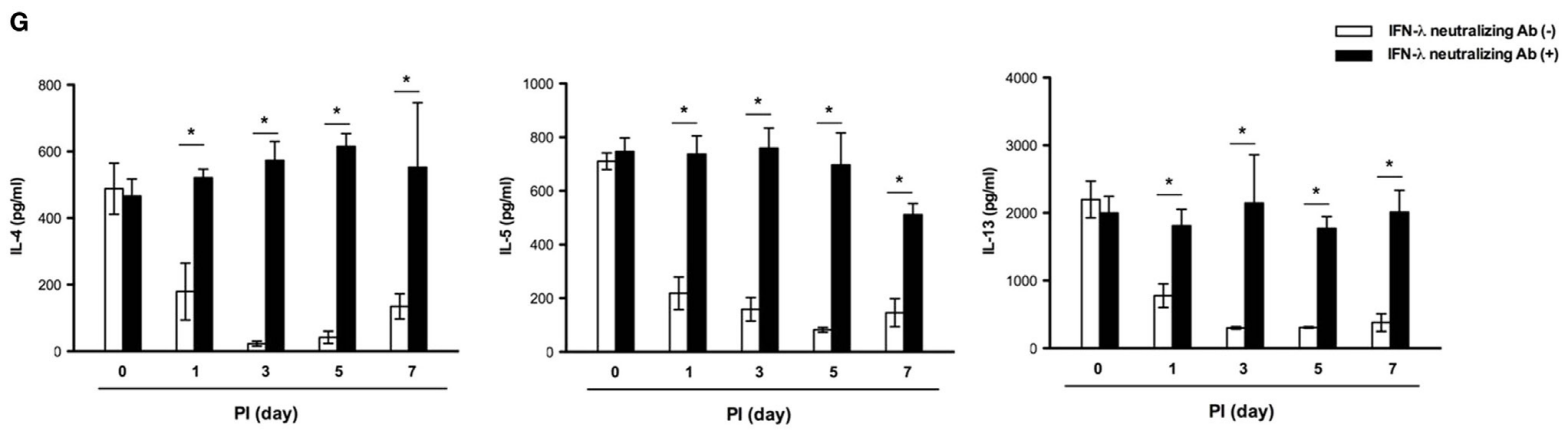
Acute influenza A virus (IAV) infection is aggravated in asthmatic mice administered neutralizing antibodies against interferon (IFN)-λ_2/3_ and IFN-γ. Asthmatic mice with isotype-control (rat IgG) antibodies were infected with 213 pfu IAV WS/33 (H1N1) (*N* = 5) and treated with neutralizing antibodies against IFN-λ_2/3_ (*N* = 5) or IFN-γ (*N* = 5). Hematoxylin/eosin (H&E)-stained micrographs were generated from lung sections obtained from IAV-infected asthmatic mice on day 7 **(A)**. Viral titer **(B)** and secreted IFN level **(C,D)** were determined by plaque assay and enzyme-linked immunosorbent assay. Histological assessment was performed using periodic acid Schiff (PAS)-stained lung sections of asthmatic mice (*N* = 5) **(E)**. Viral titer **(F)** and Th2 cytokine levels **(G)** were assessed using a plaque assay and a multiplex assay at 0 (*N* = 5), 1 (*N* = 5), 3 (*N* = 5), 5 (*N* = 5), and 7 (*N* = 5) dpi. Micrographs shown are representative of lung sections from five mice. Polymerase chain reaction, plaque assay, and multiplex assay results are presented as mean ± SD from five independent experiments (*p < 0.05 compared with the values of mice treated with IgG and neutralizing antibodies).

We next analyzed whether IFN-λ_2/3_ and IFN-γ were required to control IAV replication and whether their levels influenced host susceptibility to infection in asthmatic mice. Asthmatic mice (*N* = 5) were infected with IAV WS/33 (H1N1) *via* the intranasal route (213 pfu/30 μl) and simultaneously administered neutralizing antibodies (10 μg/30 μl) that functionally inhibit either IFN-λ_2/3_ or IFN-γ. Histological analysis of IAV-infected asthmatic mouse lung tissue showed that severe inflammation with PMN infiltration at the peribronchial areas was not observed at 7 dpi, and that the histological scores returned to normal levels. However, greater lung damage and greater mean histological scores were observed after administration of IFN-λ_2/3_- and γ-neutralizing antibodies (IAV infection: 2.6 vs. IAV + IFN-λ_2/3_-neutralizing antibodies: 12.4, IAV + IFN-γ-neutralizing antibodies: 12.5; Figure 4A). Furthermore, the viral titer in the BAL fluid was significantly higher in IAV-infected asthmatic mice that received IFN-λ_2/3_-(1.3 × 10^6^ ± 3.8 × 10^5^) and γ-neutralizing antibodies (1.4 × 10^6^ ± 2.6 × 10^5^) compared with IAV-infected asthmatic mice (2.4 × 10^4^ ± 1.6 × 10^3^) (Figure 4B).

Influenza A virus infection has been shown to incite a robust IFN-γ response in the lung, promoting the development of adaptive immunity (18). IFN-γ has been proposed to be predominantly produced by activated innate lymphoid cell type I (ILC1) and natural killer (NK) cells (19). Our current findings also showed production of IFN-γ peaked at 7 dpi in the lung tissue and BAL fluid of IAV-infected asthmatic mice and we investigated the target cells that produce IFN-γ in asthmatic mice at 7 dpi. Non-asthmatic and asthmatic mice were infected with IAV WS/33 (H1N1) (213 pfu/30 μl), and lung tissue was harvested for flow cytometry to assess the immune cells producing IFN-γ. The cytometry results showed that the percentages of IFN-γ-producing ILC1 (CD90.2^+^Lin^−^Tbet^+^IFN-γ^+^) cells (Figure S2A in Supplementary Material) and NK cells (NK1.1^+^CD3e^−^IFN-γ^+^) (Figure S2B in Supplementary Material) were not increased in the lungs of IAV-infected asthmatic mice at 7 dpi (ILC1: 24.7%, NK cell: 50.4%) compared with those of non-asthmatic mice after infection (ILC1: 54.2%, NK cell: 52.6%). However, IFN-γ-positive cells were notably observed at the bronchial epithelium in IAV-infected asthmatic mice (Figure S3 in Supplementary Material). Then, we also investigate the correlation between IFN-λ and -γ in IAV-infected asthmatic mice with administration of IFN-λ_2/3_- and γ-neutralizing antibodies. The ELISA results showed that IAV-induced IFN-λ secretion was not altered in the BAL fluid of asthmatic mice with IFN-γ-neutralizing Ab, whereas IAV-induced IFN-γ secretion (1,724.5 ± 378.5 pg/ml) was significantly reduced in asthmatic mice that were administered IFN-λ_2/3_-neutralizing antibodies before IAV infection (669.5 ± 214.6 pg/ml) (Figures 4C,D). In addition, the decreased goblet cell metaplasia and airway mucus secretion observed after IAV infection were not seen until 7 dpi in the lung tissue of IAV-infected asthmatic mice inoculated with IFN-λ_2/3_-neutralizing antibodies (Figure 4E). This finding was accompanied by higher viral titer until 7 dpi (PI 3d: 3.0 × 10^6^ + 1.3 × 10^6^, PI 5d: 3.1 × 10^6^ + 1.8 × 10^6^, PI 7d: 4.3 × 10^6^ + 1.6 × 10^5^, Figure 4F). A multiplex assay was also performed to quantify the levels of the secreted Th2 cytokines IL-4, IL-5, IL-6, and IL-13, in the BAL fluid of IAV-infected asthmatic mice with functional blocking of IFN-λ_2/3_. As noted above, secretion of Th2 cytokines was significantly attenuated in IAV-infected asthmatic mice, whereas it was not attenuated in IAV-infected mice that were inoculated with IFN-λ_2/3_-neutralizing antibodies (Figure 4G).

Overall, these results indicate that neither ILC1 nor NK cells contributed to the increased production of IFN-γ in IAV-infected asthmatic mice. They also indicate that IFN-γ is produced predominantly at the respiratory epithelium in the lungs of IAV-infected asthmatic mice. The rapid induction of IFN-λ_2/3_ could be related to the robust induction of IFN-γ at 7 days after IAV infection in respiratory epithelium of asthmatic mice; this induction seems to be central for expedite antiviral immune response and for limiting Th2 cytokine secretion after IAV infection.

### Asthmatic Mice Become Vulnerable to IAV Infection After 7 Days, but Intranasal Administration of IFN-λ_2/3_ Effectively Protects Them From IAV

**Figure 5:**
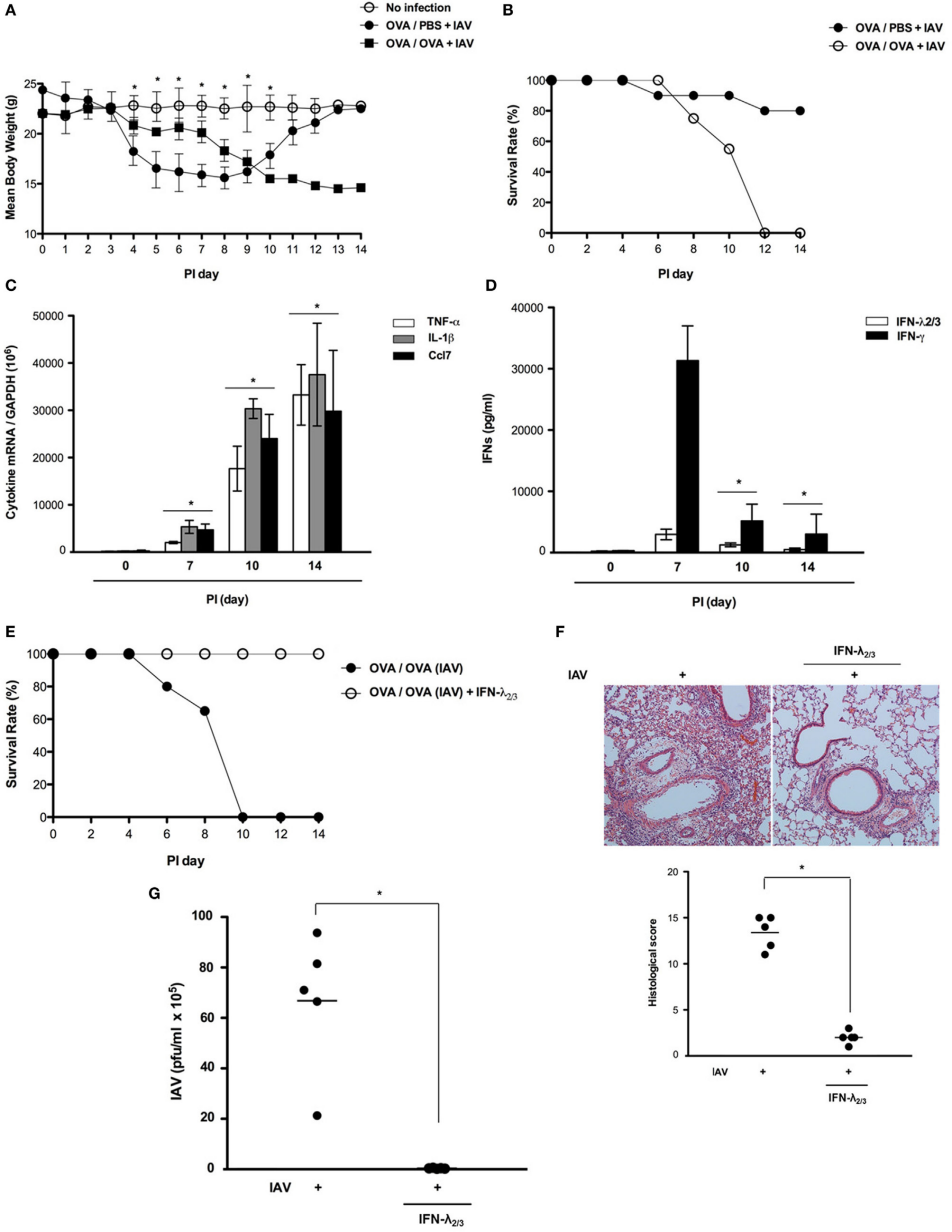
Interferon (IFN)-λ_2/3_ exerts a potent antiviral effect against influenza A virus (IAV) infection in the mouse lung. Non-asthmatic mice (*N* = 5) and asthmatic mice (*N* = 5) were infected with 213 pfu IAV WS/33 (H1N1), and body weight **(A)** and survival rate [**(B)**, *N* = 20] were assessed until 14 dpi. The mRNA levels of TNF-α, IL-1β, and Ccl7 **(C)** and the levels of secreted IFN-λ_2/3_ and IFN-γ **(D)** in the lung and bronchoalveolar lavage fluid were determined. Asthmatic mice were infected with 213 pfu IAV WS/33 (H1N1) and treated with recombinant IFN-λ_2/3_ (IFN-λ_2_: 1 μg and IFN-λ_3_: 1 μg/30 μl) (*N* = 5). As a control, the diluent phosphate-buffered saline was used (*N* = 5). Survival rate [**(E)**, *N* = 20], hematoxylin/eosin-stained lung histologic findings **(F)**, and viral titer **(G)** were determined at 14 dpi. Micrographs are representative of lung sections from five mice. Polymerase chain reaction, plaque assay, and enzyme-linked immunosorbent assay results are presented as mean ± SD from five independent experiments (*p < 0.05 compared with the values of mice with recombinant IFN-λ_2/3_).

Asthmatic mice (*N* = 5) were infected with IAV WS/33 (H1N1) *via* the intranasal route (213 pfu/30 μl), and two gross determinants of virus-induced morbidity, mean body weight and survival rate, were measured until 14 dpi. Different results were observed after 7 dpi and IAV-infected asthmatic mice showed significant weight loss from 9 dpi (Figure 5A). Specifically, noticeable morbidity was also observed in IAV-infected asthmatic mice and all of the mice were dead by 14 days after IAV infection, with a mean body weight about 15 g at the time of death (Figures 5A,B). We found that the expression levels of TNF-α, IL-1β, and Ccl7 were significantly elevated in IAV-infected asthmatic mice at 14 dpi compared with their levels at 7 dpi (Figure 5C). Moreover, IAV-infected asthmatic mice produced significantly less IFN-λ_2/3_ (PI 7d: 3,892.5 ± 643.8 pg/ml, PI 14d: 273.5 ± 81.7 pg/ml) and IFN-γ (PI 7d: 31,322.6 ± 7,318.4 pg/ml, PI 14d: 2,284.2 ± 378.5 pg/ml) (Figure 5D). Although IFN-λ_2/3_ production was driven rapidly after IAV infection in the respiratory tract of IAV-infected asthmatic mice, asthmatic mice became vulnerable to IAV infection by 7 days after infection, at which they exhibited severe lung damage.

To determine whether exogenous compensation of IFN-λ_2/3_ protects asthmatic mice from IAV infection at 14 dpi, asthmatic mice (*N* = 5) were administered recombinant IFN-λ_2/3_ (IFN-λ_2_: 1 μg, IFN-λ_3_: 1 μg) *via* the intranasal route and simultaneously inoculated with IAV WS/33 (H1N1). Interestingly, all IAV-infected asthmatic mice that received intranasal IFN-λ_2/3_ survived. Moreover, IAV-mediated histopathological lung inflammation was not observed in IFN-λ_2/3_-treated asthmatic mice, and viral titer was significantly lower in IFN-λ_2/3_-treated asthmatic mice (IAV: 6.2 × 10^6^, IAV + IFN-λ_2/3_: 1.7 × 10^3^) (Figures 5E–G). These findings suggest that the enhanced vulnerability to IAV infection of asthmatic mice could be controlled by intranasal administration of IFN-λ_2/3_ until 14 dpi.

## Discussion

This study provides a different view of the antiviral innate immune system in the asthmatic respiratory tract. Specifically, it has been generally accepted that the asthmatic airway is highly vulnerable to respiratory viral infection due to its reduced production of IFNs, which are required for clearance of viral infection and lower induction of IFNs can be involved in robust production of Th2 cytokines (10–12). Here, we showed that IFN-λ_2/3_ is induced rapidly in asthmatic mice after IAV infection, and that this cytokine is important for maintaining the antiviral immune response against IAV lung infection and for restricting Th2 cytokine secretion in asthmatic mice. These effects were observed within 7 days. Our findings also imply that intranasal administration of IFN-λ_2/3_ is a potential strategy for controlling the enhanced susceptibility of asthmatic mice to IAV infection from 7 days postchallenge onward.

Asthmatics have been reported to have increased susceptibility to respiratory viral infection including increased severity, longer duration of respiratory symptoms, and acute exacerbation of asthma (20, 21). This increased susceptibility to respiratory viral infection has been shown to be mediated by novel mechanisms including virus-induced secretion of epithelial-derived Th2 cytokines, deficient apoptosis of viral-infected cells, and lower production of IFNs after viral infection at the asthmatic airway epithelium (22, 23). In particular, IFNs are the most important component of the innate immune response and have been shown to be involved in defective innate immune responses to respiratory viral infection. The asthmatic airway epithelium appears to be profoundly deficient in viral-induced production of IFNs; reduced induction of IFNs has been shown to dampen the adaptive immune response (24–26). Although *in vivo* asthma model does not show exactly same characteristics or phenotypes as human asthma, we thought B6-oriented asthmatic model exhibit the biomedical relevance with human asthma considering histological findings, increased AHR after methacholine inhalation and peribronchial infiltration of inflammatory cells. This model could be used to investigate the susceptibility of asthmatic mice to influenza virus infection. The experiments performed here showed that asthmatic mice exhibited a significantly lower survival rate and greater loss of body weight, accompanied by higher viral titer, from 7 days after IAV lung infection onward. In addition, induction of IFN-λ_2/3_ and -γ was significantly impaired in asthmatic mouse lung tissue at 14 days after IAV infection. To the best of our knowledge, deficient innate immune responses (including reduced induction of IFNs) are expected in the asthmatic respiratory tract. Additional research aiming to understand the mechanisms driving these deficient innate immune responses is urgently required to reduce the susceptibility to viral infection of asthmatics.

While most studies have focused on exacerbation of asthma by influenza viral lung infection and the higher susceptibility to influenza virus of asthmatics, studies using a novel mouse model of asthma and influenza infection have shown that asthmatic mice are highly susceptible to influenza viral infection compared with non-asthmatic mice. Such studies are necessary for identifying novel targets for the development of effective therapies against viral infection in asthmatics (11–13).

The different hypothesis has been introduced that asthmatic mice are more resistant to influenza virus as a result of a lower viral burden in the lungs than control mice, leading to improvement of their clinical condition (14). This prompt viral clearance in asthmatic mice has been shown to be mediated by increased production of antiviral cytokines, activation of NK cells, and enhanced antigen-specific CD8+ T cell activity after infection. Other immunological mechanisms explaining the viral resistance of asthmatic mice have also been suggested that the increased survival of asthmatic mice was due to increased TGF-β-mediated tolerance to influenza infection-mediated tissue damage, rather than enhanced antiviral immunity (15). We think that it will be of interest to determine whether asthmatic mice might be also resistant to lung infection by IAV, which causes an exacerbation of asthmatic symptoms and higher mortality.

In this study, we found that asthmatic mice were also resistant to IAV infection until 7 days. Specifically, all mice survived, the mean viral titer was significantly lower, and the IAV-induced lung pathologies were reduced. Interestingly, IAV infection led to reduced Th2-related immune responses, including decreased IL-4, IL-5, and IL-13 secretion. Moreover, resolution of extensive asthma-related lung pathologies such as goblet cell hyperplasia was observed consistently in the lungs of IAV-infected asthmatic mice at 7 dpi. Based on these results, we estimate that asthmatic mice were not completely vulnerable to influenza virus infection and particularly asthmatic mice exhibited distinct immune mechanisms for resisting respiratory viral infection and restricting Th2 immunopathology in the respiratory tract.

We focused on the phase at which IFN-λ_2/3_ is rapidly induced in the lungs of IAV-infected asthmatic mice and IFN-λ_2/3_ contributes to resistance to IAV infection in asthmatic mice. In allergic airway diseases, IFN-λ cytokines are critical for driving Th1 differentiation *in vivo* and limiting Th2 and Th17 responses in the airway (7, 10). Our data also show that IFN-λ_2/3_ in particular was preferentially secreted in the lungs of IAV-infected asthmatic mice from 1 dpi and its level was maintained until 7 days. In addition, secretion of IFN-λ_2/3_ drives the transcription of IFN-γ, which was accompanied by increased viral clearance in respiratory epithelium and attenuation of Th2 cytokine secretion in IAV-infected asthmatic mice from 1 dpi onward. Interestingly, functional inhibition of IFN-λ_2/3_ in IAV-infected asthmatic mice aggravated IAV infection. Moreover, the level of Th2 cytokine and the degree of asthma-related lung histopathological findings *in vivo* were improved after IAV infection. Given these findings, the impairment of IFN-λ_2/3_ induction in response to viral infection in asthmatics has profound implications relating to the pathogenesis of virus-induced asthma exacerbation. Thus, rapid production of IFN-λ_2/3_ in the lungs of asthmatic mice in response to IAV infection could constitute the primary antiviral defense and could also restrict the secretion of Th2 cytokines. Previously, we found that the highest mRNA IFN-λ_2/3_ level and the highest level of secreted IFN-λ_2/3_ were observed at 10 dpi in the lungs of IAV-infected B6 mice (17, 27). By contrast, IFN-λ_2/3_ was released in the lungs of asthmatic mice from 1 day after IAV infection and this rapid production and maintenance of IFN-λs until 5 days after infection was accompanied by increase of IFN-γ secretion at 7 dpi. The levels of secreted IFN-β was increased by 3 dpi and the highest level of IFN-β secretion was also observed at 7 dpi in the lung of IAV-infected non-asthmatic mice (17) but IFN-β was minimally induced in the lung of IAV-infected asthmatic mice until 7 dpi. We hypothesize that the pattern of IFN secretion in response to IAV infection was altered in asthmatic mice. Specifically, asthmatic mice exhibited more rapid release of IFN-λ_2/3_, which is a central mediator of the antiviral immune response. This mechanism could explain the high resistance of asthmatic mice to respiratory viral infection at the early stage of IAV infection.

Although IFN-λ_2/3_ production was rapidly driven after IAV infection in the respiratory tract of IAV-infected asthmatic mice, asthmatic mice became vulnerable to the effect of IAV infection after 7 dpi and the levels of secreted IFN-β, IFN-λ_2/3_, and IFN-γ were completely reduced at 14 dpi in the lung of IAV-infected asthmatic mice. However, intranasal delivery of IFN-λ_2/3_ was shown to almost completely inhibit viral replication in IAV-infected asthmatic mice, as demonstrated by the significantly decreased viral titer in the lungs of mice that received intranasal IFN-λ_2/3_ compared with that of non-infected mice. In addition, the lungs of mice that received intranasal IFN-λ_2/3_ exhibited similar histological results to those of non-infected mice. Therefore, strategies involving the compensation or maintenance of IFN-λ are new opportunities for invoking an effective antiviral defense against IAV infection. IFN-λ is thus a promising new target for reducing susceptibility to respiratory viral infection in asthmatics.

In summary, rapid induction of IFN-λ is a distinctive immunologic finding in IAV-infected asthmatic mice and this effect is accompanied by reduced initial viral spread in asthmatic lung. This rapid induction of IFN-λ is crucial for controlling viral load and for maintaining effective antiviral immune mechanisms in asthmatics at early stage of infection. Our study provides compelling evidence that IFN-λ could have therapeutic potential for treating IAV-related respiratory infection in asthmatics.

## Ethics Statement

All experiments were approved by the Institutional Review Board of Seoul National University College of Medicine (IRB number 2015-2642) and were carried out in accordance to LABORATORY ANIMAL ACT of Korean Ministry of Food and Drug Safety for enhancing the ethics and reliability on animal testing through appropriate administration of laboratory animals and animal testing.

## Author Contributions

Conception and design: SA, YJ, and HJK. Designed research: YJ, AJ, YH, HL, and HYK. Analyzed experimental data and performed the data interpretation including flow cytometry: SC and HJK. Drafted the manuscript for important intellectual content: SC and HJK.

## Conflict of Interest Statement

The authors declare that the research was conducted in the absence of any commercial or financial relationships that could be construed as a potential conflict of interests.

## Supplementary Material

Figure S1 Transpulmonary resistance in IAV-infected asthmatic mice. Airway hyper-responsiveness was measured as methacholine-induced increases in transpulmonary resistance in mechanically ventilated mice. Data are expressed as mean value of percentage increased from base line of the transpulmonary resistance ± SD values of three mice (White dot: OVA/OVA, Black dot:OVA/PBS, Black square:IAV-infected OVA/OVA).

Figure S2 Flow cytometric analysis for ILC1 and NK cells. The percentage of IFN-γ produced ILC1 (Tbet+Lineage-IFN-γ+) **(A)** and NK cells **(B)** (NK1.1+CD3e-IFN-γ+) were evaluated in the lung tissue of non-asthmatic (OVA/PBS) and asthmatic mice (OVA/OVA) before and after IAV infection.

Figure S3 Immunohistochemistry for IFN-γ in non-asthmatic and asthmatic mice. Immunohistochemical analysis of IFN-γ using DAB chromogen was performed in lung sections from non-asthmatic (OVA/PBS) and asthmatic mice (OVA/OVA) before and after IAV infection. The number of IFN-γ-positive cells was significantly increased in the lung tissue of IAV-infected asthmatic mice and bronchial epithelial cells showed strong expression of IFN-γ (Original magnification x200).

## References

[1] Umetsu DT DeKruyff RH. The regulation of allergy and asthma. Immunol Rev (2006) 212:238–55. 10.1111/j.0105-2896.2006.00413.x 16903918

[2] Kubo M. Innate and adaptive type 2 immunity in lung allergic inflammation. Immunol Rev (2017) 278:162–72. 10.1111/imr.12557 28658559

[3] Castillo JR Peters SP Busse WW Asthma exacerbations: pathogenesis, prevention, and treatment. J Allergy Clin Immunol Pract (2017) 5:918–27. 10.1016/j.jaip.2017.05.001 28689842 PMC5950727

[4] Holt PG Strickland DH. Interactions between innate and adaptive immunity in asthma pathogenesis: new perspectives from studies on acute exacerba-tions. J Allergy Clin Immunol (2010) 125:963–72. 10.1016/j.jaci.2010.02.011 20394979

[5] Garcia-Sastre A Biron CA Type 1 interferons and the virus-host relationship: a lesson in detente. Science (2006) 312:879–82. 10.1126/science.1125676 16690858

[6] Cheon H Holvey-Bates EG Schoggins JW Forster S Hertzog P Imanaka N IFNbeta-dependent increases in STAT1, STAT2, and IRF9 mediate resistance to viruses and DNA damage. EMBO J (2013) 32:2751–63. 10.1038/emboj.2013.203 24065129 PMC3801437

[7] Galani IE Triantafyllia V Eleminiadou EE Koltsida O Stavropoulos A Manioudaki M Interferon-lambda mediates non-redundant front-line antiviral protection against influenza virus infection without compromising host fitness. Immunity (2017) 46:875–90. 10.1016/j.immuni.2017.04.025 28514692

[8] Jewell NA Cline T Mertz SE Smirnov SV Flaño E Schindler C Lambda interferon is the predominant interferon induced by influenza A virus infection in vivo. J Virol (2010) 84:11515–22. 10.1128/JVI.01703-09 20739515 PMC2953143

[9] Kim BJ Cho SW Jeon YJ An S Jo A Lim JH Intranasal delivery of Duox2 DNA using cationic polymer can prevent acute influenza A viral infection in vivo lung. Appl Microbiol Biotechnol (2018) 102:105–15. 10.1007/s00253-017-8512-1 28936773

[10] Contoli M Ito K Padovani A Poletti D Marku B Edwards MR Th2 cytokines impair innate immune responses to rhinovirus in respiratory epithelial cells. Allergy (2015) 70:910–20. 10.1111/all.12627 25858686

[11] Message SD Laza-Stanca V Mallia P Parker HL Zhu J Kebadze T Rhinovirus-induced lower respiratory illness is increased in asthma and related to virus load and Th1/2 cytokine and IL-10 production. Proc Natl Acad Sci U S A (2008) 105:13562–7. 10.1073/pnas.0804181105 18768794 PMC2528869

[12] Contoli M Message SD Laza-Stanca V Edwards MR Wark PA Bartlett NW Role of deficient type III interferon-lambda production in asthma exacerbations. Nat Med (2006) 12:1023–6. 10.1038/nm1462 16906156

[13] Koltsida O Hausding M Stavropoulos A Koch S Tzelepis G Ubel C IL-28A (IFN-lambda2) modulates lung DC function to promote Th1 immune skewing and suppress allergic airway disease. EMBO Mol Med (2011) 3:348–61. 10.1002/emmm.201100142 21538995 PMC3377081

[14] Ishikawa H Sasaki H Fukui T Fujita K Kutsukake E Matsumoto T. Mice with asthma are more resistant to influenza virus infection and NK cells activated by the induction of asthma have potentially protective effects. J Clin Immunol (2012) 32:256–67. 10.1007/s10875-011-9619-2 22134539 PMC3305878

[15] Furuya Y Furuya AK Roberts S Sanfilippo AM Salmon SL Metzger DW Prevention of influenza virus-induced immunopathology by TGF-beta produced during allergic asthma. PLoS Pathog (2015) 11:e1005180 10.1371/journal.ppat.1005180 26407325 PMC4583434

[16] Patel DA You Y Huang G Byers DE Kim HJ Agapov E Interferon response and respiratory virus control are preserved in bronchial epithelial cells in asthma. J Allergy Clin Immunol (2014) 134:1402–12. 10.1016/j.jaci.2014.07.013 25216987 PMC4261010

[17] Kim S Kim MJ Kim CH Kang JW Shin HK Kim DY The superiority of IFN-lambda as a therapeutic candidate to control acute influenza viral lung infection. Am J Respir Cell Mol Biol (2017) 56:202–12. 10.1165/rcmb.2016-0174OC 27632156

[18] Verhoeven D Perry S Pryharski K. Control of influenza infection is impaired by diminished interferon-γ secretion by CD4 T cells in the lungs of toddler mice. J Leukoc Biol (2016) 100:203–12. 10.1189/jlb.4A1014-497RR 26823488 PMC6608081

[19] Dahl ME Dabbagh K Liggitt D Kim S Lewis DB. Viral-induced T helper type 1 responses enhance allergic disease by effects on lung dendritic cells. Nat Immunol (2004) 5:337–43. 10.1038/ni1041 14973436

[20] Steinke JW Borish L. Immune responses in rhinovirus-induced asthma exacerbations. Curr Allergy Asthma Rep (2016) 16:78. 10.1007/s11882-016-0661-2 27796793 PMC5797654

[21] Toussaint M Jackson DJ Swieboda D Guedán A Tsourouktsoglou TD Ching YM Host DNA released by NETosis promotes rhinovirus-induced type-2 allergic asthma exacerbation. Nat Med (2017) 23:681–91. 10.1038/nm.4332 28459437 PMC5821220

[22] Jarjour NN Esnault S Interleukin-33: a potential link between rhinovirus infections and asthma exacerbation. Am J Respir Crit Care Med (2014) 190:1336–7. 10.1164/rccm.201411-1949ED 25496100 PMC4299653

[23] Johnston SL. Innate immunity in the pathogenesis of virus-induced asthma exacerbations. Proc Am Thorac Soc (2007) 4:267–70. 10.1513/pats.200701-030AW 17607011

[24] Tworek D Heroux D O’Byrne SN Mitchell P O’Byrne PM Denburg JA. Toll-like receptor-induced expression of epithelial cytokine receptors on haemopoietic progenitors is altered in allergic asthma. Clin Exp Allergy (2017) 47:900–8. 10.1111/cea.12913 28252235

[25] Riese RJ Finn PW Shapiro SD Influenza and asthma: adding to the respiratory burden. Nat Immunol (2004) 5:243–4. 10.1038/ni0304-243 14985710

[26] Sykes A Edwards MR Macintyre J del Rosario A Bakhsoliani E Trujillo-Torralbo MB Rhinovirus 16-induced IFN-alpha and IFN-beta are deficient in bronchoalveolar lavage cells in asthmatic patients. J Allergy Clin Immunol (2012) 29:1506–14. 10.1016/j.jaci.2012.03.044 22657407

[27] Hong SN Kim JY Kim H Kim DY Won TB Han DH Duox2 is required for the transcription of pattern recognition receptors in acute viral lung infection: an interferon-independent regulatory mechanism. Antiviral Res (2016) 134:1–5. 10.1016/j.antiviral.2016.08.017 27546489

