# Supplementary figures and images for: Initial Influenza Virus Replication Can Be Limited in Allergic Asthma Through Rapid Induction of Type III Interferons in Respiratory Epithelium
Source: Front Immunol. 2018 May 17;9:986. doi: 10.3389/fimmu.2018.00986 (PMC5966536; doi:10.3389/fimmu.2018.00986)

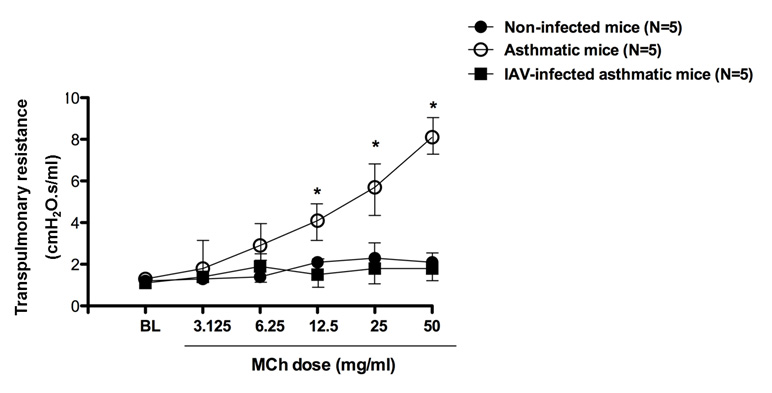

Supplement: Figure S1 — Transpulmonary resistance in IAV-infected asthmatic mice. Airway hyper-responsiveness was measured as methacholine-induced increases in transpulmonary resistance in mechanically ventilated mice. Data are expressed as mean value of percentage increased from base line of the transpulmonary resistance ± SD values of three mice (White dot: OVA/OVA, Black dot:OVA/PBS, Black square:IAV-infected OVA/OVA). [file image_1.JPEG]

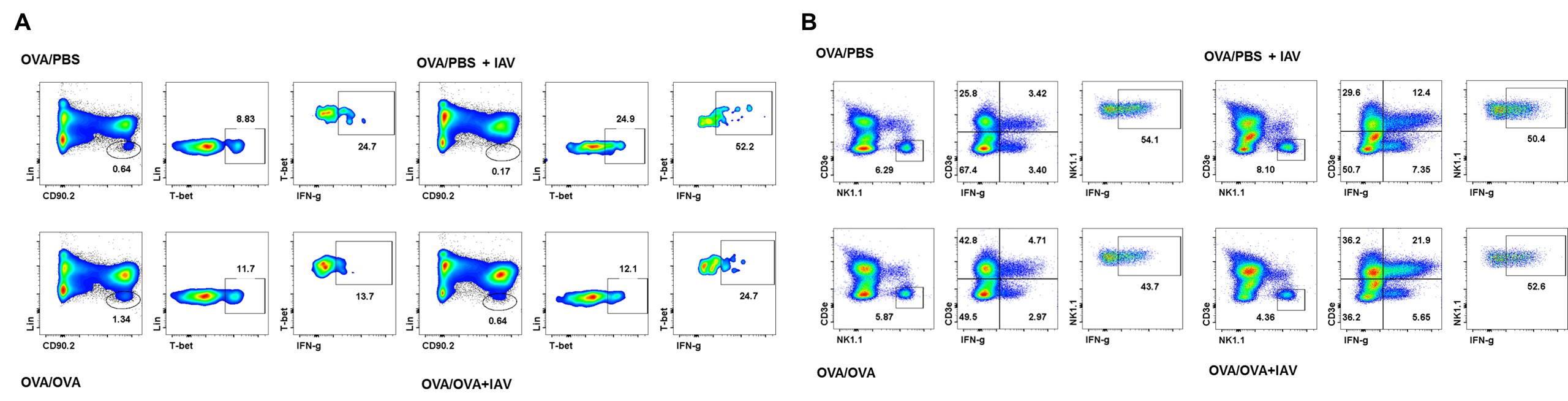

Supplement: Figure S2 — Flow cytometric analysis for ILC1 and NK cells. The percentage of IFN-γ produced ILC1 (Tbet+Lineage-IFN-γ+) (A) and NK cells (B) (NK1.1+CD3e-IFN-γ+) were evaluated in the lung tissue of non-asthmatic (OVA/PBS) and asthmatic mice (OVA/OVA) before and after IAV infection. [file image_2.JPEG]

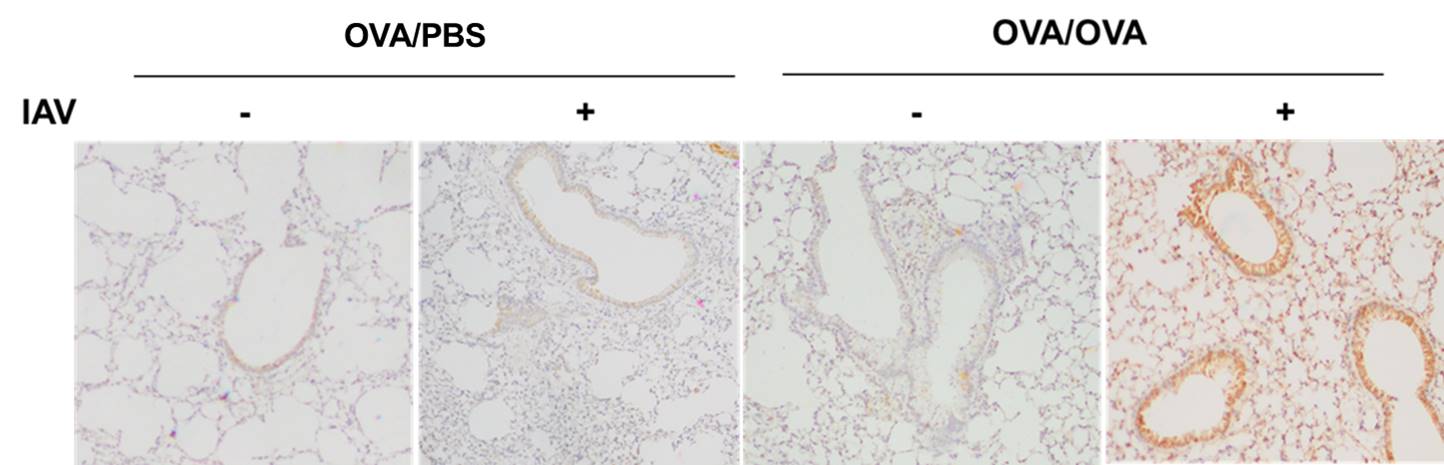

Supplement: Figure S3 — Immunohistochemistry for IFN-γ in non-asthmatic and asthmatic mice. Immunohistochemical analysis of IFN-γ using DAB chromogen was performed in lung sections from non-asthmatic (OVA/PBS) and asthmatic mice (OVA/OVA) before and after IAV infection. The number of IFN-γ-positive cells was significantly increased in the lung tissue of IAV-infected asthmatic mice and bronchial epithelial cells showed strong expression of IFN-γ (Original magnification x200). [file image_3.JPEG]
